# Knowledge Level of Undescended Testis in Saudi Arabia: Why Are We Facing Delayed Presentation?

**DOI:** 10.7759/cureus.42226

**Published:** 2023-07-20

**Authors:** Shahad T Abdulrahman, Maryam Dahlawi, Mansour M Almalki, Bassam M Bin Laswad, Rahaf G Baaqeel, Hazem M Aljabri, Mohammed H Ageel

**Affiliations:** 1 College of Medicine, Umm Al-Qura University, Makkah, SAU; 2 Department of Surgery, Umm Al-Qura University, Makkah, SAU

**Keywords:** pediatrics, infant, saudi arabia, infertility, orchidopexy, cryptorchidism, undescended testis

## Abstract

Background

Undescended testis (UDT) or cryptorchidism is a common pediatric surgical presentation. The accepted time for surgical correction (orchidopexy) is when the patient is aged from six months, and should ideally be completed before one year of age. In Saudi Arabia, the median age at the time of orchidopexy is 25 months, exceeding the recommended surgery time.

Objective

The objective of the study was to determine the factors that cause delayed presentation of UDT among children in Saudi Arabia.

Methods

A cross-sectional, nationwide study targeting the general population of Saudi Arabia. The study was conducted in November 2022 using a validated questionnaire distributed through social media platforms.

Results

A total of 2360 participants were enrolled. Over half (54.92%) had not heard about UDT. Further, 48.5% of the participants did not know the age of UDT presentation, and 49.1% had no idea about the treatment modality. Moreover, 13.9% had known someone diagnosed with UDT, while 17.68% discovered UDT after more than a year. In addition, 1.5% had previous experience with UDT, and 22.86% were diagnosed after more than a year. There was a significantly high level of knowledge among participants who had experienced UDT. The three most common reasons for delaying the intervention for UDT patients were a lack of community awareness of UDT, parents' ignorance and neglect, and a lack of early screening programs (22.3%, 21.7%, and 19.7%, respectively).

Conclusion

Our data demonstrated a significant lack of awareness of UDT among the Saudi population since 1296 (54.92%) of the participants had not heard about UDT. The presence of such an awareness gap necessitates cultural education about the topic of UDT by all capable facilities, including medical schools, hospitals, and primary healthcare centers.

## Introduction

Undescended testis (UDT), also known as cryptorchidism, is the most common congenital urogenital defect among boys. It is referred to as the failure of one or both testicles to descend into the normal scrotal position by the time of birth [1]. The incidence of UDT in full-term infants or birth weight >2.5 kg is estimated to be 4.6%, while the incidence in premature infants or birth weight <2.5 kg ranges from 1.1% to 45.3% [2]. Prematurity, a family history of UDT, and underlying congenital abnormalities are possible risk factors for UDT [3].

Normally, spontaneous testicular descent occurs in the first six months of life due to a surge in gonadotropins and androgenic hormones. Thereafter, the probability of spontaneous descent decreases [4,5]. There is a three- to tenfold increased risk of developing malignant changes in cases of UDT compared to infants with descended testes. Restoring the testicles to the normal position will not decrease the risk of malignant changes, although it will stop the process of degeneration [6]. UDT is associated with several other adverse outcomes, such as infertility, testicular torsion, and inguinal hernia [4,5]. Early recognition and correct referral of UDT cases are crucial to prevent delayed management and consequently several complications [7].

Updated guidelines recommend screening for UDT within three days of birth, at six to eight weeks of age, and at four to five months of age [8]. In consideration of the fact that there is no spontaneous descent of the testes after the age of six months, the patient should be referred to an appropriate surgical specialist for further evaluation and surgical correction (orchidopexy) [8].

A study conducted in Germany revealed a strong positive association between the risk of infertility and the delay in surgical intervention [9]. In Saudi Arabia, the median age group at the time of orchidopexy is 25 months, meaning the procedure takes place outside the recommended timeframe [1]. Among the Saudi population in Hail, only 29.9% of patients meet the recommended timeframe for orchidopexy. A statistically significant difference has been found in the awareness of UDT when there is previous experience of UDT, namely, either having a child or knowing a relative with UDT [10].

According to one study conducted in Saudi Arabia, the most reported reason for delayed orchidopexy is a late referral to a pediatric surgeon [11]. Since the delay in UDT presentation gives rise to serious complications, it is important to determine the causative factors delaying such presentation to fix and prevent them. Accordingly, we should expand studies to other cities in Saudi Arabia to determine these factors and knowledge of UDT at the community level, as knowing the factors and assessing the level of knowledge to enhance them can help minimize delayed presentation.

Given the foregoing, we conducted this study to determine and measure the factors that cause the delayed presentation of UDT in children and the level of knowledge of UDT among the community in Saudi Arabia.

## Materials and methods

Study design and participants

A nationwide cross-sectional study was conducted in Saudi Arabia in November 2022. The study enrolled Saudi Arabian residents who agreed to participate and excluded those under 18 years of age.

Sample size

The survey was created in Google Forms (Google, Mountain View, California) and distributed through social media platforms. We used the OpenEpi website, version 3.01, to estimate the sample size, keeping the confidence interval at 95%; the result was 385 participants [12]. However, as this is a community-based study, we aimed for a more extensive study sample to improve accuracy and enhance generalizability. Overall, we were able to collect 2360 responses.

Study tool

We adopted a valid questionnaire from a previously published article and added a question to assess knowledge of the complications caused by an intervention delay [10]. The questionnaire was translated into the Arabic language by native speakers. A group of expert researchers and a pediatric surgery consultant assessed the questionnaire's reliability and content validity. A pilot study was conducted, and minor changes were made. The pilot study data were excluded from the final results.

The questionnaire of this study included four sections. The first section covered the participants' demographic characteristics. The respondents were asked about their previous experience with UDT in the second section. The third section assessed the general knowledge of UDT. Finally, the last section assessed the participants' awareness of the treatment options of UDT and potential complications.

Data analysis

Categorical data were presented as frequencies and percentages, and numerical data as means and standard deviations. The Chi-squared test was used to compare the categorical variables, and a p-value of ≤0.05 was considered to be statistically significant. The data analysis was carried out using Statistical Package for Social Sciences (SPSS) version 23 (IBM Inc., Armonk, New York,).

Ethical considerations

This study was approved by the Biomedical Ethics Committee at the College of Medicine of Umm Al-Qura University, Makkah, Saudi Arabia (approval number: HAPO-02-K-012-2022-11-1232). Electronic informed consent was obtained from each participant before starting the questionnaire. 

## Results

A total of 2360 participants were involved in this study; most of them were Saudis (89%), more than half were females (62.6%), while (37.3%) were males. Approximately 54.4% of the studied sample were 18-25 years and 24.3% were 26-35 years. Of the participants, 58.4% were single, 37.1% were married, while the minority (3%) were divorced or widowed (1.4%). Regarding educational differences, 65.4% had a Bachelor's degree, while 19.9% and 9.1% had a high school education and diploma, respectively. In addition, 43.4% of the participants were students, whereas 32.8% were employees. Altogether, 34.3% had children, and 65.7% did not. Two-thirds of the participants were not healthcare providers (Table 1).

**Table 1:** Demographic characteristics of study participants (n= 2360)

Variable	No	%
Nationality		
Saudi	2102	89.0%
Non-Saudi	258	10.9%
Gender		
Male	881	37.3%
Female	1479	62.6%
Age (years)		
18-25 years	1282	54.4%
26-35 years	574	24.3%
36-45 years	261	11.1%
46-55 years	186	7.8%
56 or more years	57	2.4%
Marital status		
Single	1379	58.4%
Married	877	37.1%
Divorced	71	3.0%
Widow	33	1.4%
Educational level		
Less than high school	45	1.9%
High school	469	19.9%
Diploma	215	9.1%
Bachelor's	1544	65.4%
Postgraduate degree (Master's, PhD)	87	3.7%
Occupation		
Employee	775	32.8%
Unemployed	239	10.1%
Student	1025	43.4%
Housewife	321	13.6%
Do you have children?		
Yes	810	34.3%
No	1550	65.7%
Are you a healthcare provider?		
Yes	780	33.1%
No	1580	66 .6%

**Figure 1:**
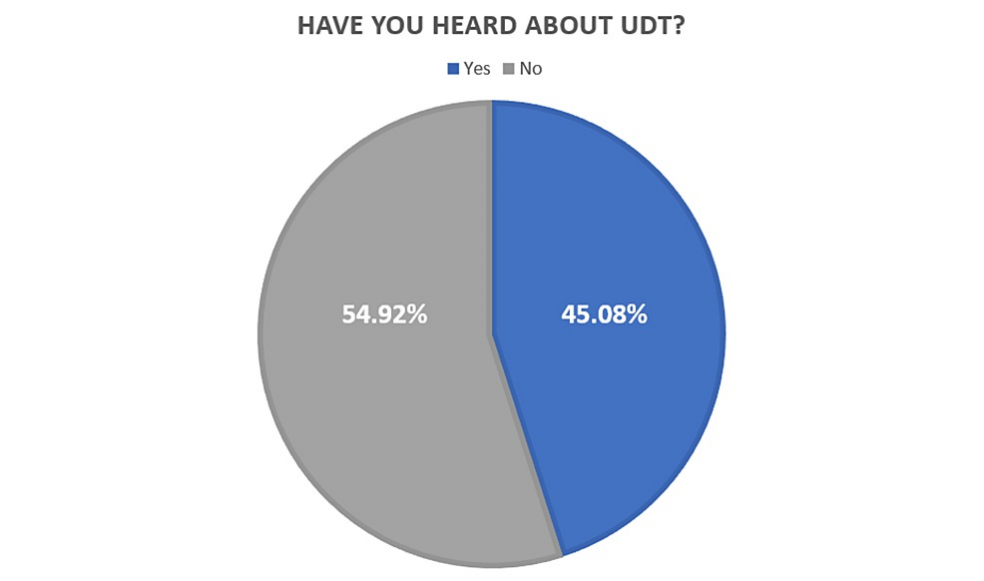
Percentage distribution of participants who have heard about undescended testis before (n=2360) UDT - undescended testis

In regard to the participants' knowledge of UDT, as shown in Figure 1, almost half (n=1296, 54.92%) had never heard about UDT compared to those that had heard about it (n=1064, 45.08%). Of the respondents, 47.3% agreed that UDT can be present from birth, 4.2% said from adolescence, and 48.5% did not know. When the respondents were asked if UDT can affect testicular function, 54.8% believed it could, while 4% believed it could not, and 41.1% did not know. Moreover, 43.4% were convinced UDT has complications, whereas 3.6% thought it does not, while 53% did not know. Furthermore, 49.1% had no idea about the treatment modality for UDT, 42.7% agreed that surgical intervention is the treatment modality for UDT, 3.9% and 2.5% chose hormonal therapy and oral medication, respectively, and 1.9% thought that it needs no treatment. More than half of the respondents (55.8%) did not know the optimum time for surgical intervention; 14.3% considered it to be during the first five months, 14.1% said six months to one year, 8.9% said from 1 year, and 6.9% stated soon after birth. Additionally, 65.9% presumed that there are benefits from early treatment, while 32.5% did not know if there are any benefits. When the participants were asked about the complications of delaying UDT intervention, 81.1% believed that it could cause testicular atrophy, 75.2% considered infertility as one of the complications, 67% thought it might lead to testicular torsion, and 63.4% agreed that malignancy could arise from UDT (Table 2).

**Table 2:** Participants' knowledge regarding undescended testis (n=2360)

Questions	No	%
At which age does undescended testis arise?		
Since birth	1116	47.3%
Adolescences age	99	4.2%
I don't know	1145	48.5%
Does undescended testis affect the testicular function?		
Yes	1295	54.8%
No	94	4.0%
I don't know	971	41.1%
Does undescended testis has any complications?		
Yes	1025	43.3%
No	84	3.6%
I don't know	1251	53.0%
What is the treatment of undescended testis?		
Oral medication	58	2.5%
Hormone therapy	92	3.9%
Surgical intervention	1007	42.7%
Does not need treatment	44	1.9%
I don't know	1159	49.1%
What is the optimum time of surgery?		
After birth	162	6.9%
First 5 months	338	14.3%
6 months - 1 year	332	14.1%
One year and more	210	8.9%
I don't know	1318	55.8%
Is there any benefit from early treatment?		
Yes	1556	65.9%
No	38	1.6%
I don't know	766	32.5%
Do you think testicular atrophy is a potential harm of delaying the intervention?		
Yes	1916	81.1%
No	444	18.8%
Do you think infertility is a potential harm of delaying the intervention?		
Yes	1775	75.2%
No	585	24.8%
Do you think testicular torsion is a potential harm of delaying the intervention?		
Yes	1581	67.0%
No	779	33.0%
Do you think malignancy risk is a potential harm of delaying the intervention?		
Yes	1498	63.4%
No	862	4.2%

**Figure 2:**
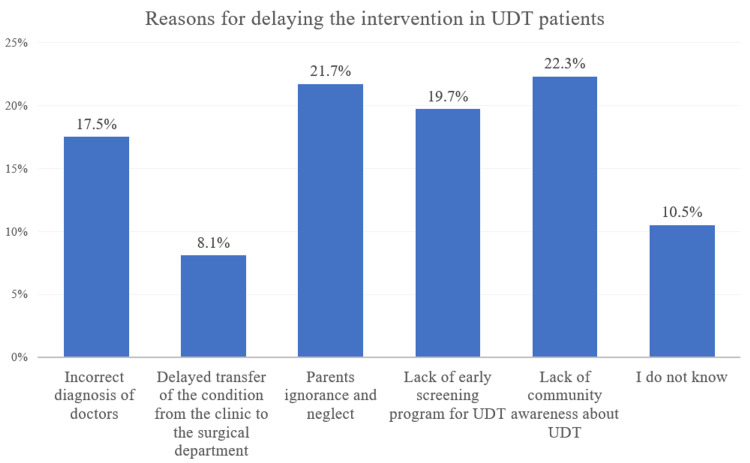
Reasons of delaying the intervention in undescended testis patients (n=2360) UDT - undescended testis

Interestingly, as shown in Figure 2, the most common reasons from the participants' perspectives about delaying intervention in UDT patients included a lack of community awareness of UDT (22.3%) and parents' ignorance and neglect (21.7%). Moreover, 19.7% blamed the absence of an early screening program for UDT, 17.5% considered the inaccurate diagnosis of doctors as a reason, 8.1% thought it was due to the delayed transfer of the condition from the clinic to the surgical department, and 10.5% did not know.

In total, 363 participants had experience with UDT (15.4%). As shown in Table 3, 35 (1.5%) of them had been diagnosed with UDT, while 328 (13.9%) had gained experience from knowing a friend or relative diagnosed with UDT (Table 4). A total of 28.1% were diagnosed directly after birth, 20.4% were diagnosed in the first three months of life, 9.1% at four to five months, 7.7% at six months to one year, and 18.2% after one year. Half of the participants (50.1%) were diagnosed by physicians, 30% discovered their UDT at home, 6.6% of cases were noticed by others (e.g., circumcision clinic, relative, and nanny), and 13.2% did not know who discovered it. The vast majority (89.8%) followed up with a doctor, while 4.7% did not follow up, and 5.5% did not know if they followed up. On the type of intervention, 63.4% had surgical intervention, 6.1% mentioned medical intervention, 10.5% had other interventions, and 20% did not know. 

**Table 3:** Characteristics of participants who have been diagnosed with undescended testis (n=35 ) UDT - undescended testis

Variables	No	%
Have you experienced UDT before?		
Yes	35	1.5%
No	2325	98.5%
Discovered time		
Diagnosed directly after birth	13	37.14%
In the first 3 months of life	6	17.14%
4-5 months	2	5.71%
6 months to 1 year	6	17.14%
More than 1 year	8	22.86%
Discovered by who?		
Diagnosed by the physician	20	57.14%
Discovered at home by parents	9	25.71%
Noticed by others (circumcision Clinic, relatives, and nannies)	3	8.57%
I don't know	3	8.57%
Follow up with doctor		
Yes	29	82.86%
No	5	14.29%
I don't know	1	2.86%
Type of intervention		
Medical	6	17.14%
Surgical	18	51.43%
Other intervention	5	14.29%
I don't know	6	17.14%

**Table 4:** Characteristics of participants' relatives and friends diagnosed with undescended testis (n=328) UDT - undescended testis

Variables	No	%
Have your children, relatives or friends ever been diagnosed with UDT?		
Yes	328	13.9%
No	2032	86.1%
Discovered time		
Diagnosed directly after birth	89	27.13%
In the first 3 months of life	68	20.73%
4- 5 months	31	9.45%
6 months to 1 year	22	6.71%
More than 1 year	58	17.68%
I don't know	60	18.29%
Discovered by who?		
Diagnosed by the physician	162	49.39%
Discovered at home by parents	100	30.49%
Noticed by others (circumcision clinic, relatives, and nannies).	21	6.4%
I don't know	45	13.72%
Follow up with doctor		
Yes	297	90.55%
No	12	3.66%
I don't know	19	5.79%
Type of intervention		
Medical	16	4.88%
Surgical	212	64.63%
Other intervention	33	10.06%
I don't know	67	20.43%

Table 5 presents the association between knowledge level and participants who had been diagnosed with UDT (n=35), as well as participants who knew someone with this condition (n=328). Regarding the age at which UDT arises, the majority of the participants in both groups answered the question correctly, which was "since birth" (65.71% and 71.65%, respectively, as it has a significant p-value (p<0.001). For the second question, 77.14% of the participants with a history of UDT and 68.9% of the participants who knew someone with UDT knew that UDT affects testicular function with a significant p-value (p<0.001). In terms of the treatment of UDT, 54.29% of the participants in the first group identified that it requires surgical intervention, which is the correct answer, and 20% did not know. Similarly, in the second group, 68.6% answered surgical intervention, and 21.24% did not know, with a significant p-value (p<0.001). In regard to the complications of UDT, 77.14% of the participants with a history of UDT and 59.76% of the participants in the other group were aware that UDT has complications with a significant p-value (P=<0.001). Moreover, the majority of the participants in the first group (28.57%) answered that the optimum time for surgery is between six months and one year, which is the correct answer, and 22.86% did not know. Of the participants who knew someone with UDT, only 21.65% answered correctly, and many (29.57%) did not know, followed by 22.87% who responded in the first five months with a significant p-value (p<0.001). Most of the participants in both groups were aware of the benefits of the early treatment of UDT (82.86% and 82.93%, respectively, with a significant p-value (p<0.001). In terms of knowledge of the complications of delaying intervention, of the participants with a history of UDT, 82.82% identified that UDT leads to testicular atrophy, 74.29% infertility, 77.14% testicular torsion, and 74.29% the risk of malignancy. In the other group, 83.54% identified that UDT leads to testicular atrophy, 73.48% infertility, 69.82% testicular torsion, and 65.55% the risk of malignancy.

**Table 5:** Association between participants' experience of undescended testis and knowledge level UDT - undescended testis *significant p-value (<0.05)

Variables	Have been diagnosed with UDT(n=35)			Knew someone diagnosed with UDT (n=328)		
	No	%	p-value	No	%	p-value
At which age did undescended testis arise?			<0.001*			<0.001*
Since birth	23	65.71%		235	71.65%	
Adolescences age	6	17.14%		10	3.05%	
I don't know	6	17.14%		83	25.3%	
Does undescended testis affect the testicular function?			<0.001*			<0.001*
Yes	27	77.14%		226	68.9%	
No	2	5.71%		23	7.01%	
I do not know	6	17.14%		79	24.09%	
Does undescended testis has any complications?			<0.001*			<0.001*
Yes	27	77.14%		196	59.76%	
No	2	5.71%		23	7.01%	
I don't know	6	17.14%		109	33.23%	
What is the treatment of undescended testis?			<0.001*			<0.001*
Oral medication	4	11.43%		12	3.66%	
Hormone therapy	1	2.86%		10	3.05%	
Surgical intervention	19	54.29%		225	68.6%	
Does not need treatment	4	11.43%		11	3.35%	
I don't know	7	20%		70	21.24	
What is the optimum time of surgery?			<0.001*			<0.001*
After birth	5	14.29%		25	7.62%	
First 5 months	7	20%		75	22.87%	
6 months to 1 year	10	28.57%		71	21.65%	
One year and more	5	14.29%		60	18.29%	
I don't know	8	22.86%		97	29.57%	
Is there any benefit from early treatment?			<0.001*			<0.001*
Yes	29	82.86%		272	82.93%	
No	4	11.43%		7	2.14%	
I don't know	2	5.71%		49	14.94%	
Do you think testicular atrophy is a potential harm of delaying the intervention?			0.258			0.117
Yes	29	82.86%		274	83.54%	
No	6	17.14%		54	16.46%	
Do you think infertility is a potential harm of delaying the intervention?			0.692			0.775
Yes	26	74.29%		241	73.48%	
No	9	25.71%		87	26.52%	
Do you think testicular torsion is a potential harm of delaying the intervention?			0.078			0.077
Yes	27	77.14%		229	69.82%	
No	8	22.86%		99	30.18%	
Do you think malignancy risk is a potential harm of delaying the intervention?			0.097			0.137
Yes	26	74.29%		215	65.55%	
No	9	25.71%		113	34.45%	

The results for the knowledge of the participants who had heard about UDT (n=1064) and those who had not (n=1296) are shown in Table 6. Regarding the age of the presentation of UDT, in the group of participants who had heard about UDT, most (78.57%) responses were correct, namely that UDT begins to arise from birth. By contrast, in the other group, most of the participants did not know (82.95%), with a significant p-value (p<0.001). Of those participants who had heard about UDT, 77.35% were aware that UDT affects testicular function, and 17.48% did not know. In the group who had not heard about UDT, only 28.7% responded correctly, and more than half (68.29%) did not know the correct answer with a significant p-value (p<0.001). Additionally, in the first group, 70.96% believed that UDT can cause complications, and 24.72% did not know, whereas only 20.83% answered correctly and 76.23% did not know in the other group with a significant p-value (p<0.001). In terms of treatment, in the group of participants who had heard about UDT, the majority (72.65%) recognized that it requires surgical intervention, and 15.88% did not know, while in the other group of participants who had not heard about UDT, the majority of the respondents (76.39%) did not know, and only 18.06% recognized that it requires surgical intervention with a significant p-value (p<0.001). Regarding the optimum time of surgical intervention, which is six months to one year, of the participants who had heard about UDT, 24.25% identified the correct answer, and 30.73% did not know. In the second group, most of the participants (76.47%) did not know, and only 5.71% identified the correct answer with a significant p-value (p<0.001). Most of the participants in the first group (86.75%) were aware that there are benefits from early treatment, while the most frequent response in the second group (57.18%) was "I don't know" with a significant p-value (p<0.001). Moreover, regarding the potential harm of delaying intervention in UDT patients, of the group of participants who had heard about UDT, 87.12% identified that it could lead to testicular atrophy, 81.95% infertility, 69.36% testicular torsion, and 66.92% malignancy, whereas in the other group who had not heard about this condition, 76.31% identified testicular atrophy, 69.68% infertility, 65.05% testicular torsion, and 60.65% risk of cancer.

**Table 6:** Association between participants' who had heard about undescended testis and those who had not, and knowledge level UDT - undescended testis *significant p-value (<0.05)

Variables	Heard about UDT (n=1064)			Has not heard about UDT (n=1296)		
	No	%	p-value	No	%	p-value
At which age did undescended testis arise?			<0.001*			<0.001*
Since birth	836	78.57%		180	13.89%	
Adolescences age	58	5.45%		41	3.16%	
I don't know	170	15.98%		1075	82.95%	
Does undescended testis affect the testicular function?			<0.001*			<0.001*
Yes	823	77.35%		372	28.7%	
No	55	5.17%		39	3.01%	
I don't know	186	17.48%		885	68.29%	
Does undescended testis has any complications?			<0.001*			<0.001*
Yes	755	70.96%		270	20.83%	
No	46	4.32%		38	2.93%	
I don't know	263	24.72%		988	76.23%	
What is the treatment of undescended testis?			<0.001*			<0.001*
Oral medication	31	2.91%		27	2.08%	
Hormone therapy	59	5.55%		33	2.55%	
Surgical intervention	773	72.65%		234	18.06%	
Does not need treatment	32	3.01%		12	0.93%	
I don't know	169	15.88%		990	76.39%	
What is the optimum time of surgery?			<0.001*			<0.001*
After birth	100	9.4%		62	4.78%	
First 5 months	221	20.77%		117	9.03%	
6 months to 1 year	258	24.25%		74	5.71%	
One year and more	158	14.85%		52	4.01%	
I don't know	327	30.73%		991	76.47%	
Is there any benefit from early treatment?			<0.001*			<0.001*
Yes	923	86.75%		533	41.13%	
No	16	1.5%		22	1.7%	
I don't know	125	11.75%		741	57.18%	
Do you think testicular atrophy is a potential harm of delaying the intervention?			<0.001*			<0.001*
Yes	927	87.12%		989	76.31%	
No	137	12.88%		307	23.69%	
Do you think infertility is a potential harm of delaying the intervention?			<0.001*			<0.001*
Yes	872	81.95%		903	69.68%	
No	192	18.05%		393	30.32%	
Do you think testicular torsion is a potential harm of delaying the intervention?			<0.001*			<0.001*
Yes	738	69.36%		843	65.05%	
No	326	30.64%		453	34.95%	
Do you think malignancy risk is a potential harm of delaying the intervention?			<0.001*			<0.001*
Yes	712	66.92%		786	60.65%	
No	352	33.08%		510	39.35%	

## Discussion

UDT is one of the most common congenital abnormalities found among males; it is present in 1 to 4.5% of newborns, with a higher incidence in preterms (30-45%). It requires early recognition and correct referral for proper management because any delay in correction is associated with an increased risk of several adverse outcomes, such as testicular cancer, infertility, testicular torsion, and inguinal hernia.

The purpose of this study was to determine and measure the factors that cause the delayed presentation of UDT in children and the knowledge level of UDT among community members in Saudi Arabia. According to the research results, a majority of the study participants were women (62.6%), were not employed in the healthcare sector (66.6%), and the majority (80%) had education above high school level. However, despite their education level, only 56% of the participants were aware of the UDT condition. These demographic features are similar to a study conducted in Hail, Saudi Arabia [10], where the majority of participants were also women (63.6%) with a Bachelor's degree (80.2%).

In our study, a small percentage (15.4%) of participants had previous experience with UDT; those with prior experience had a better understanding of the condition. This is consistent with a study in Hail [10], where 23.1% of participants had prior experience. Among participants in our study with previous experience, most had been diagnosed with UDT shortly after birth; these findings align with the Hail study [10], where 22.4% of cases were diagnosed at birth. However, another study in Nigeria [13] found no patients diagnosed at birth, while a study in Riyadh [14] found 45% of patients diagnosed after age one, which contrasts with our findings. In our study, the majority of UDT conditions were discovered by physicians. This is consistent with the Hail study [10], as 47.9% of their participants were diagnosed by a physician, as well as with an Australian study [15] in which 90.1% of their children were diagnosed by pediatricians and 4.8% by general physicians. Furthermore, the Hail study [10] showed that 56% of their participants were operated surgically; this is similar to our findings, as our participants who have been diagnosed with UDT and those with relatives and friends diagnosed with UDT said that the type of intervention was surgical (51.4% and 64.6%, respectively).

The knowledge of community members plays a significant role in presenting the condition to healthcare providers as early as possible; thus, in our study, we determined the level of our participants' knowledge. Over half of our participants (54.9%) had not heard of UDT and lacked knowledge about diagnosis, complications, treatment, and surgery timing. The Hail [10] study showed similar findings regarding awareness but had higher awareness about complications. Those with prior experience had better knowledge, as confirmed by a significant association between experience and knowledge level (p<0.001). Furthermore, we reported that participants who had heard of UDT had better knowledge than those who had not, with a significant association between awareness and knowledge level (P<0.001). This is similar to the study in Hail [10], which found a similar association (p=0.0004). Our study found community awareness, parental ignorance/neglect, and lack of early screening programs to be common reasons for delayed presentation of UDT, while the Hail study [10] identified delayed transfer to the surgical department as the primary reason.

A delay in intervention causes many complications, such as inguinal hernia, infertility, torsion, and a three- to tenfold increased risk of malignancy [4-6]. We demonstrated over half of our sample had never heard of UDT, highlighting a significant lack of awareness that requires further efforts to improve public knowledge. This can be achieved through various means, such as educating new parents about common anomalies and raising awareness in schools, health centers, and hospitals; improved knowledge can facilitate faster detection and earlier presentation, reducing the likelihood of high-risk complications. Screening programs and training for primary care providers are also necessary for early detection and referral to specialists. However, our study's self-reported data and focus on only one congenital anomaly are limitations, indicating the need for further research on awareness of congenital anomalies.

## Conclusions

There is a significant lack of awareness of UDT among the Saudi population because 1296 (54.92%) of the participants had not heard about UDT. The three most nominated reasons for delaying the intervention in UDT patients were a lack of community awareness of UDT, parents' ignorance and neglect, and a lack of early screening programs for UDT (22.3%, 21.7%, and 19.7%, respectively). The presence of such an awareness gap necessitates cultural education about the topic of UDT by all capable facilities, including medical schools, hospitals, and primary healthcare centers.
